# A Predictive Framework for Integrating Disparate Genomic Data Types Using Sample-Specific Gene Set Enrichment Analysis and Multi-Task Learning

**DOI:** 10.1371/journal.pone.0044635

**Published:** 2012-09-13

**Authors:** Brian D. Bennett, Qing Xiong, Sayan Mukherjee, Terrence S. Furey

**Affiliations:** 1 Departments of Statistical Science, Computer Science, and Mathematics, Institute for Genome Sciences and Policy, Duke University, Durham, North Carolina, United States of America; 2 Department of Genetics, Department of Biology, Lineberger Comprehensive Cancer Center, and Carolina Center for Genome Sciences, The University of North Carolina at Chapel Hill, Chapel Hill, North Carolina, United States of America; National Taiwan University, Taiwan

## Abstract

Understanding the root molecular and genetic causes driving complex traits is a fundamental challenge in genomics and genetics. Numerous studies have used variation in gene expression to understand complex traits, but the underlying genomic variation that contributes to these expression changes is not well understood. In this study, we developed a framework to integrate gene expression and genotype data to identify biological differences between samples from opposing complex trait classes that are driven by expression changes and genotypic variation. This framework utilizes pathway analysis and multi-task learning to build a predictive model and discover pathways relevant to the complex trait of interest. We simulated expression and genotype data to test the predictive ability of our framework and to measure how well it uncovered pathways with genes both differentially expressed and genetically associated with a complex trait. We found that the predictive performance of the multi-task model was comparable to other similar methods. Also, methods like multi-task learning that considered enrichment analysis scores from both data sets found pathways with both genetic and expression differences related to the phenotype. We used our framework to analyze differences between estrogen receptor (ER) positive and negative breast cancer samples. An analysis of the top 15 gene sets from the multi-task model showed they were all related to estrogen, steroids, cell signaling, or the cell cycle. Although our study suggests that multi-task learning does not enhance predictive accuracy, the models generated by our framework do provide valuable biological pathway knowledge for complex traits.

## Introduction

A fundamental challenge in genomics is discovering and understanding the molecular and genetic basis of complex traits. A deeper understanding of complex traits will potentially lead to a better diagnosis and treatment of complex diseases. A number of studies have used gene expression assays to model, at a molecular level, direct influences driving phenotypic variation. A shortcoming of this approach is that gene expression differences may be driven by many genomic and environmental factors, including underlying genetic variation [1]. In this study, we developed a framework to integrate complementary evidence of differential expression and genotype variation associated with a complex phenotype. Results based on this framework aim to uncover pathways that influence phenotype with biologically relevant differences, specifically differential gene expression, genetic variation, or a combination of the two. Pathways showing both expression and genetic differences suggest that transcriptional variation may be driven in part by genetic variation. The goal of this framework is to better model the genetic and molecular causes of complex traits, including complex diseases.

Several previous efforts have explored integrating different genomic data types [2]–[11]. Many have focused on using gene expression and DNA copy number data. For example, one study modeled gene expression based on the copy number variation for genes on the same chromosome arm [2]. Others looked for regions with high copy number alterations and then searched for important genes within these regions [3]–[6]. In one study focused on colorectal cancer, they calculated fold changes in expression and copy number data between normal and diseased samples, ordered all probes based on chromosomal location, and then searched for large chromosomal segments showing coordinated expression and copy number changes [3]. This analysis revealed many regions with copy number gain or loss along with differential expression of genes in the region, and they identified several candidate genes in regions of interest for further study.

Other studies searched for significant differences in individual genes for multiple genomic data [7]–[9]. For example, one study integrated gene expression, copy number, DNA methylation, and loss of heterozygosity (LOH) data for breast cancer [7]. They looked for genes that had significant changes in all of these data types when compared to normal. This analysis revealed that *ERBB2*, an important breast cancer gene, simultaneously showed amplification, loss of heterozygosity, loss of methylation, and a drastic increase in gene expression.

Genome-wide association studies generally utilize genotype data by itself, but a few studies have integrated this genotype data with other genomic data. One study integrated genotype data with gene expression data for various cancer types to find genes with expression changes driven by genotype differences [10]. They selected cancer-associated genes whose expression profiles are known to predict treatment outcome and looked for genotype patterns within these genes. They created a model in which expression profiles and genotype patterns for selected genes were combined and used to predict the treatment success of prostate and breast cancer patients.

Some studies performed a pathway-level integrative analysis [3], [8], [11]. For example, one study integrated gene expression and somatic mutation data to identify pathways frequently altered in prostate cancer [3]. A single tumor was considered to have an altered pathway if one or more genes in the pathway had a somatic mutation or had an expression level that was significantly different than in normal prostate. Pathways altered in a large percentage of the samples were considered frequently altered. This study identified three well-known cancer pathways as frequently altered: PI3K, RAS/RAF, and RB.

Our study provides a new framework for integrating different genomic data types that consists of two key steps, sample-specific pathway analysis and multi-task learning, that individually have proven useful in classification analyses but have never been used together. Most previous integrative approaches performed sequential or independent analyses of each data type. Our method differs from these approaches in that genome-wide expression and genotype data, encoded as pathway enrichment scores, are simultaneously used to build the final predictive model. This eliminates the restriction of using results from one independent analysis to filter results from the other, and instead allows the model to equally and simultaneously consider data from each experiment.

Gene set analysis explores biological data in the context of pathways. This approach examines the simultaneous enrichment of multiple genes belonging to particular pathways, in contrast to single-gene analysis, which searches for differences in individual genes. For complex traits, many phenotypic differences are associated with perturbations in specific pathways [12]–[15]. Also, pathway analysis provides results that are highly reproducible between studies [16], [17]. Many methods have been developed to analyze data on the gene set level [16], [18]–[21]. In general, these provide a single measure of enrichment for each gene set across all samples. In order to use multi-task learning to build a predictive model, sample-specific pathway enrichment information is required. To obtain this information, our framework extends a gene set enrichment software package called ASSESS (Analysis of Sample Set Enrichment ScoreS) [22], which provides a measure of pathway enrichment for each sample.

Although gene set enrichment analysis can improve the interpretability of results [23] and increase predictive performance [22], an additional advantage of our pathway analysis step is providing a method to more easily integrate data types that may have very different structure. For example, expression data consists of gene-based continuous values, whereas genotype data consists of discrete SNP-based genotypes. By first obtaining sample-specific gene set enrichment scores for each data type, this also acts as a normalization step to allow each data type to be combined with other data types.

Multi-task learning [24] is a supervised learning approach to building predictive models from data that contain complementary information. While other supervised learning methods perform well when there is a single data type, studies have shown an improved performance in predictive accuracy in some instances when simultaneously building multiple models from data with related information [24]–[26]. Our framework builds predictive models using the sample-specific enrichment scores from ASSESS for different data types. Multi-task learning provides a way to integrate these data types as different tasks in the model. Our framework uses regularized multi-task learning [25], which is a Support Vector Machine (SVM) [27] implementation of multi-task learning. Multi-task learning aims to take advantage of data with similar information between tasks while also incorporating information unique to each task. In the context of our pathway-based multi-task framework, similar information means similar pathway enrichment among data types, whereas different information means pathway enrichment that is unique to a data type.

In order to examine the ability of multi-task learning to simultaneously utilize similar and different pathway enrichment properties in our study, we compared the predictive ability of multi-task learning to single-task learning and a concatenated data learning model. Single-task learning independently builds separate models for each task and does not consider whether there is similar or different information between tasks. In this study, we performed single-task learning by independently using a standard SVM to build a predictive model for each data type. A concatenated data model combines all data together by simply concatenating it into a single data set to take advantage of all information together, but it does not distinguish which task the information originated from. In this study, we built concatenated data models by combining all of the enrichment scores for all data types together into a single data set and used a standard SVM to build a single predictive model.

Multi-task learning builds a model that attempts to take advantage of the strengths of both single-task learning and concatenated data models. It does this by calculating a common effect shared among all tasks (see Methods), similar to a concatenated data model. At the same time, it determines a task-specific effect that is unique to each task (see Methods), similar to a single-task model. Successful multi-task learning models should show an improvement in predicative performance when compared to a single-task model and a concatenated data model. The framework we describe here can integrate several different types of genomic data with each sample having been assigned to one of two phenotypic classes, along with a collection of gene sets. It uses this to produce a predictive model that can also identify gene sets important in distinguishing phenotype.

In this study, we examine the performance of this framework under a variety of conditions, and determine how useful this framework is for genomic data. Although our framework can be used to integrate many different genomic data types, this study focuses on the integration of gene expression and genotype data. Incorporating genotype data required the development of a novel method for obtaining sample-specific enrichment scores for this data. To test the performance of our framework, we generated simulated data and compared the predictive accuracy of multi-task learning to single-task learning and a concatenated data model. Results show that multi-task learning has a similar predictive accuracy as the single-task learning and concatenated data models. We also show that models that consider all tasks, such as multi-task or concatenated data models, are better at discovering gene sets with pathways containing genes that are both differentially expressed and genetically associated with a phenotype. We also used our framework to explore differences between estrogen receptor (ER) positive and negative breast cancer. The top 15 gene sets from the multi-task model were involved with estrogen, steroids, cell signaling, or the cell cycle.

## Results

**Figure 1:**
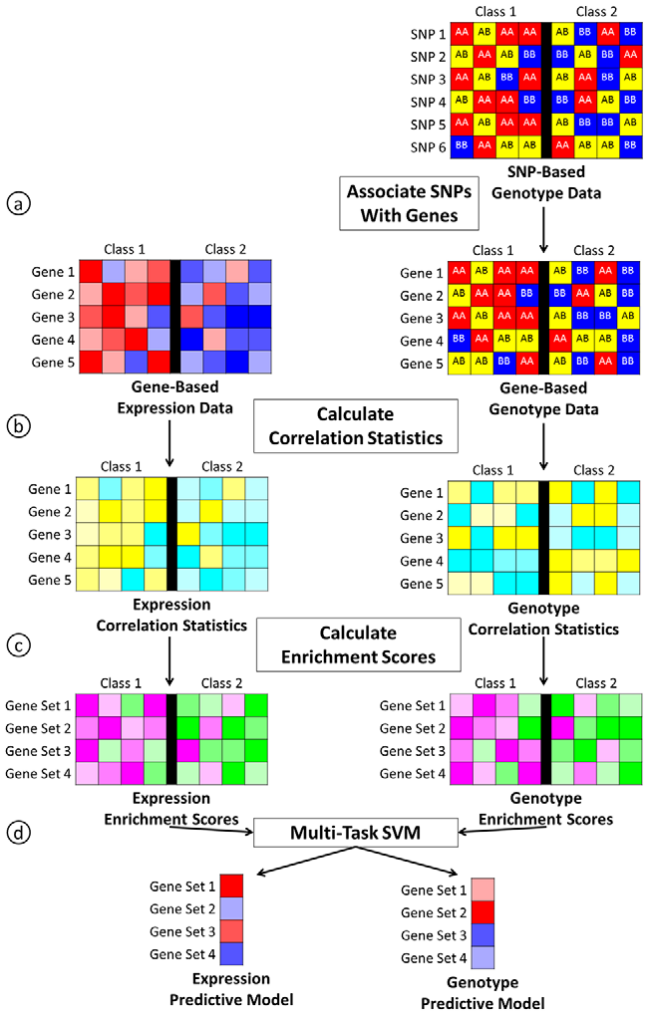
Overview of the multi-task pipeline. For genotype data, we associate each gene with a single SNP (a). Next, we calculate correlation statistics using the gene-based data for each data type (b). We then calculate enrichment scores using the correlation statistics for each data type (c). Finally, we build a predictive model for each data type in an integrative way using the enrichment scores for each data type and a multi-task SVM (d). In this overview, ASSESS corresponds to steps b and c.

An overview of the analysis pipeline for integrating gene expression and genotype data within our framework is presented in Figure 1. The two key steps are first a sample-based analysis on the pathway level using ASSESS (Figure 1b–1c), and second the integration of genomic data into a predictive model using a multi-task SVM (Figure 1d).

In step one, ASSESS takes as input gene-based genomic data for samples belonging to one of two phenotypic classes. It then produces sample-specific enrichment scores for a collection of gene sets. To do this, it first calculates a correlation statistic for each gene in each sample that represents the degree to which the gene-based data matches the summary profile of that gene in samples from one phenotype class compared to the other (Figure 1b). Then, it ranks all genes based on this correlation statistic for each sample and uses gene set enrichment analysis to determine the enrichment of pathways within samples (Figure 1c).

**Figure 2:**
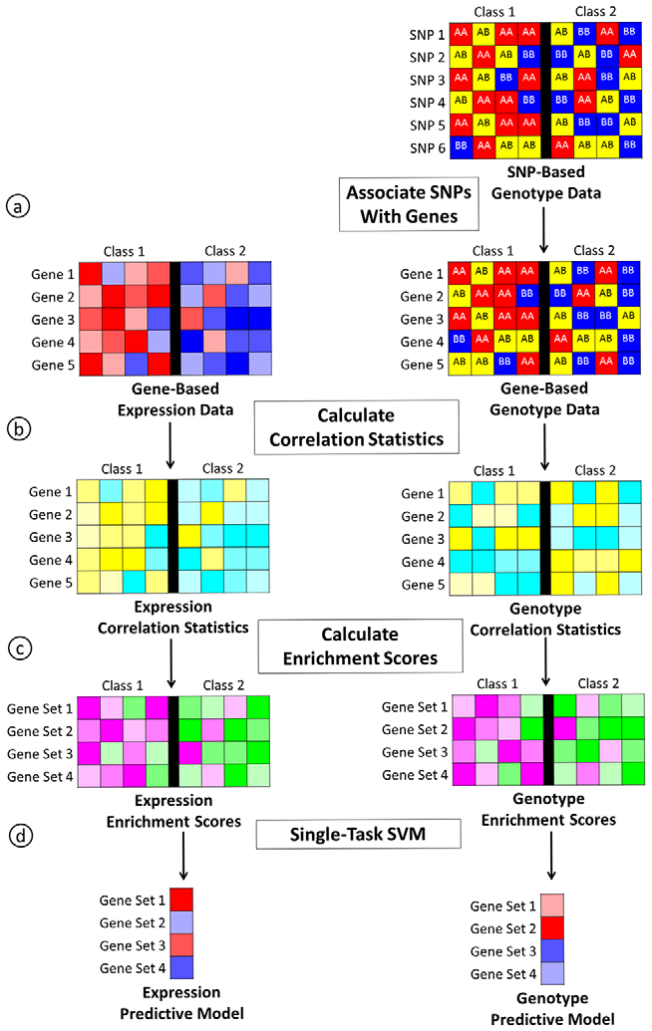
Overview of the single-task pipeline. For genotype data, we associate each gene with a single SNP (a). Next, we calculate correlation statistics using the gene-based data for each data type (b). We then calculate enrichment scores using the correlation statistics for each data type (c). Finally, we independently build a predictive model for each data type using the enrichment scores for each data type and a standard SVM (d). In this overview, ASSESS corresponds to steps b and c.

**Figure 3:**
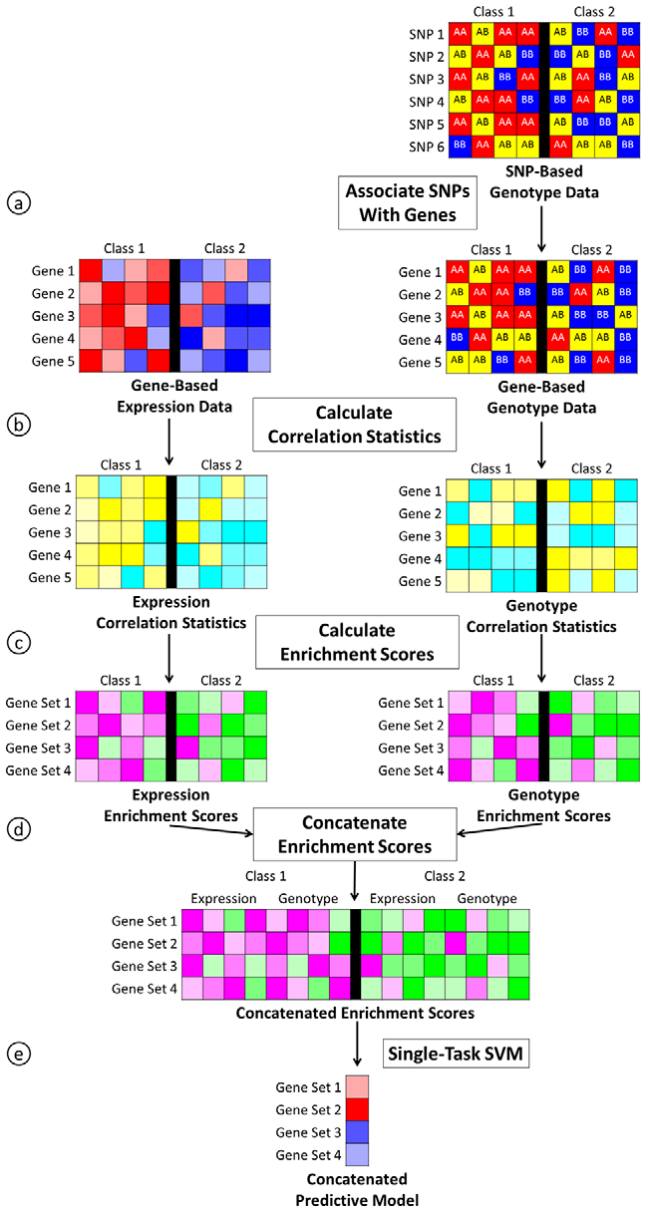
Overview of the concatenated data pipeline. For genotype data, we associate each gene with a single SNP (a). Next, we calculate correlation statistics using the gene-based data for each data type (b). We then calculate enrichment scores using the correlation statistics for each data type (c). We next concatenate the enrichment scores for all data types into a single data set (d). Finally, we build a single predictive model using the concatenated enrichment scores and a standard SVM (e). In this overview, ASSESS corresponds to steps b and c.

In step two, we use the enrichment scores from ASSESS that are calculated independently for several different data types as the input tasks to the multi-task model (Figure 1d). Multi-task learning assumes that the samples among the different tasks are independent, and it does not require that the different data come from the same matched samples or that there are the same number of samples in each task. In this study, we compare the performance of multi-task models with single-task and concatenated data models. The single-task model uses the enrichment scores from ASSESS to build separate single-task models for each data type (Figure 2). The concatenated model combines the enrichment scores from all data types and builds a single model from this concatenated data set (Figure 3).

Since these methods do not treat matched data, meaning expression and genotype data are from the same samples, differently from unmatched data, they may fail utilize important information if the data is matched. To address this, we explored models that specially consider multiple data from matched samples that produce a single prediction for each sample. First, we used a *summed prediction* model that sums the predictions from the single-task models for each data type to obtain a single prediction for each sample. Second, we created a *summed enrichment score* model that sums the ASSESS enrichment scores for each gene set in each sample and uses these summed enrichment scores within a single-task model to obtain a single prediction. Third, we used a *merged* model that takes enrichment scores from ASSESS for each data type for a given sample and merges them into a single feature vector of enrichment scores for that sample. We used these merged enrichment scores with a single-task model to obtain a single prediction.

ASSESS was previously developed for use with gene expression data, but not genotype data. Therefore, we first extended ASSESS to obtain sample-specific enrichment scores for genotype data. We next evaluated the performance of our framework by simulating multiple data sets to explore the following questions: 1) does the similarity of tasks influence the predictive performance of a multi-task model; 2) does the number of samples impact the predictive performance; 3) does the number of tasks influence the predictive performance; and 4) does an integrated approach improve our ability to discover pathways that are enriched in several data types. Finally, we applied our framework to a breast cancer data set to analyze differences between ER+ and ER- samples.

### Obtaining enrichment scores for genotype data

To facilitate the integration of genotype data with gene expression, we extended the previously developed software package ASSESS to obtain sample-specific gene set enrichment scores for genotype data. A key challenge in using any gene-based method for the analysis of genotype data is mapping the SNP-based data to the gene level. We designed our framework to select a single SNP to represent each gene (Figure 1a). For all SNPs located within a predefined distance surrounding and including a gene, we performed a Pearson's chi-square test on each SNP to determine its correlation with phenotype and selected the SNP with the highest correlation as the representative SNP for that gene.

After mapping the genotype data to the gene level, we calculated the ASSESS correlation statistic for each sample and gene (Figure 1b) by comparing the genotype for a sample and gene to the genotypes of all samples in each class for that gene (see Methods). We used these correlation statistics within the ASSESS software to then obtain gene set enrichment scores (Figure 1c). Using ASSESS for gene expression and genotype data, we obtain similar sample-specific enrichment scores for both data types that can be integrated in a multi-task analysis.

### Predictive performance of multi-task with varying similarity of tasks

Multi-task learning may offer an advantage when there is a balance of similar and different pathway enrichment among different data types. If there is too much similar enrichment, then multi-task learning may not outperform a concatenated data model. If the enrichment is too different, then a multi-task model may not outperform single-task learning. To test the performance of our multi-task framework, we created a simulation to compare the predictive accuracy of a multi-task model to a single-task and concatenated data model with varying similarity in the tasks.

We simulated gene expression and genotype data with gene sets belonging to one of the following gene set types:

1. 10 genes that were differentially expressed between the two phenotype classes and genetically associated with phenotype;
2. 10 genes that were differentially expressed but not genetically associated;
3. 10 genes that were not differentially expressed, but genetically associated;
4. 10 genes that were neither differentially expressed nor genetically associated.

We generated data for 5 experimental scenarios, each with a varied number of gene sets from each gene set type (Table 1). Data with gene sets predominantly from gene set type 1 have similar enrichment across tasks, while data predominantly from gene set type 2 and 3 have different enrichment across tasks.

**Table 1:** Scenarios with varying similarity between tasks.

	Type 1 Gene Sets	Type 2 Gene Sets	Type 3 Gene Sets	Type 4 Gene Sets
**Scenario 1**	0 (0/0)	20 (20/0)	20 (0/20)	60 (0/0)
**Scenario 2**	5 (5/5)	15 (15/0)	15 (0/15)	65 (0/0)
**Scenario 3**	10 (10/10)	10 (10/0)	10 (0/10)	70 (0/0)
**Scenario 4**	15 (15/15)	5 (5/0)	5 (0/5)	75 (0/0)
**Scenario 5**	20 (20/20)	0 (0/0)	0 (0/0)	80 (0/0)

Values in parenthesis represent the number of gene sets with genes that are differentially expressed and the number of gene sets with genes that are genetically associated, respectively. Scenario 1 contains data with most different enrichment between data types; scenario 5 contains data with most similar enrichment.

For each scenario, we simulated matched expression and genotype data for 50 training samples, which were equally split into 2 phenotypes. The data was matched such that the expression level of a gene for a given sample was generated taking into account the genotype of the SNP associated with that gene for that sample (see Methods). We used our multi-task framework to train predictive models with these samples. Then, we used these models to obtain predictions for 50 test samples as to which phenotypic class they belong to. We also used the same data and ASSESS-based enrichment scores to evaluate single-task SVMs and an SVM with the expression and genotype enrichment scores concatenated. In addition, we used the same enrichment scores to evaluate the summed prediction, summed enrichment score, and merged models, which utilize matched data. We repeated this procedure 200 times to obtain 10,000 predictions for each scenario and calculated the percentage of correct predictions for each scenario and SVM model type (Tables 2, 3, and 4).

**Table 2:** Performance of expression data with varying levels of similarity.

	Single-Task	Multi-Task	Concatenated
**Scenario 1**	59.58%±0.52%	58.98%±0.50%	58.69%±0.49%
**Scenario 2**	59.58%±0.52%	59.01%±0.48%	59.06%±0.50%
**Scenario 3**	59.58%±0.52%	59.17%±0.50%	59.20%±0.48%
**Scenario 4**	59.58%±0.52%	59.25%±0.50%	59.34%±0.49%
**Scenario 5**	59.58%±0.52%	59.55%±0.50%	59.26%±0.49%

Percentage of correct predictions with standard error for the expression data using single-task, multi-task, and concatenated models with varying levels of similarity in the data.

**Table 3:** Performance of genotype data with varying levels of similarity.

	Single-Task	Multi-Task	Concatenated
**Scenario 1**	69.26%±0.47%	66.00%±0.48%	62.33%±0.53%
**Scenario 2**	69.26%±0.47%	66.14%±0.44%	63.23%±0.50%
**Scenario 3**	69.26%±0.47%	66.58%±0.45%	63.91%±0.50%
**Scenario 4**	69.26%±0.47%	66.87%±0.46%	64.53%±0.48%
**Scenario 5**	69.26%±0.47%	67.76%±0.46%	65.18%±0.49%

Percentage of correct predictions with standard error for the genotype data using single-task, multi-task, and concatenated models with varying levels of similarity in the data.

**Table 4:** Performance of matched data models with varying levels of similarity.

	Summed Prediction	Summed Enrichment Score	Merged
**Scenario 1**	67.97%±0.52%	67.87%±0.46%	71.19%±0.47%
**Scenario 2**	67.97%±0.52%	67.99%±0.44%	71.19%±0.47%
**Scenario 3**	67.97%±0.52%	68.11%±0.46%	71.19%±0.47%
**Scenario 4**	67.97%±0.52%	68.68%±0.48%	71.19%±0.47%
**Scenario 5**	67.97%±0.52%	68.53%±0.49%	71.19%±0.47%

Percentage of correct predictions with standard error using summed prediction, summed enrichment score, and merged models with varying levels of similarity in the data.

For the expression data, the predictive performance was similar for all scenarios and model types (Table 2). For the genotype data, multi-task learning had a significant improvement in predictive accuracy compared to the concatenated model for all scenarios, but failed to perform better than the single-task model (Table 3). Also, accuracy improved for the multi-task and concatenated models as the scenarios contained more similar enrichment (Table 3). For the models that utilize matched data, the summed prediction and summed enrichment score models failed to perform better than the best unmatched model, but the merged model had a significant improvement in predictive accuracy compared to the best unmatched model (Table 4). Although the difference in predictive accuracy was statistically significant in some cases, the actual predictive performance was similar in these instances.

### Predictive performance of multi-task with varying number of samples

We next determined the effect that sample size has on our multi-task framework when compared to a single-task or concatenated data model. To do this, we first simulated matched expression and genotype data using gene sets from scenario 3. In the previous analysis, we used 50 samples to train the model. In this analysis, we varied the number of training samples from 10 to 200. As above, we used the training samples to build a multi-task, single-task, and concatenated data model, and we simulated an equal number of test samples to generate predictions. We also evaluated the summed prediction, summed enrichment score, and merged models, which utilize matched data. We repeated to obtain 10,000 predictions for each number of samples and calculated the percentage of correct predictions for each number of samples and each type of model (Tables 5, 6, and 7).

**Table 5:** Performance of expression data with varying sample sizes.

	Single-Task	Multi-Task	Concatenated
**10 Samples**	56.47%±0.46%	55.85%±0.47%	55.72%±0.48%
**20 Samples**	58.15%±0.53%	58.33%±0.51%	58.26%±0.51%
**50 Samples**	59.10%±0.54%	59.02%±0.50%	59.16%±0.50%
**100 Samples**	61.00%±0.49%	61.26%±0.47%	61.20%±0.53%
**200 Samples**	63.67%±0.54%	63.10%±0.56%	62.74%±0.54%

Percentage of correct predictions with standard error for the expression data using single-task, multi-task, and concatenated models with varying sample sizes.

**Table 6:** Performance of genotype data with varying sample sizes.

	Single-Task	Multi-Task	Concatenated
**10 Samples**	55.10%±0.49%	56.06%±0.47%	56.02%±0.48%
**20 Samples**	62.52%±0.49%	62.93%±0.47%	62.05%±0.48%
**50 Samples**	70.84%±0.47%	69.02%±0.45%	65.11%±0.51%
**100 Samples**	76.50%±0.45%	74.10%±0.48%	70.28%±0.54%
**200 Samples**	82.50%±0.53%	80.19%±0.50%	74.91%±0.56%

Percentage of correct predictions with standard error for the genotype data using single-task, multi-task, and concatenated models with varying sample sizes.

**Table 7:** Performance of matched data models with varying sample sizes.

	Summed Prediction	Summed Enrichment Score	Merged
**10 Samples**	59.19%±0.48%	58.57%±0.49%	59.20%±0.48%
**20 Samples**	63.45%±0.50%	63.86%±0.49%	64.03%±0.52%
**50 Samples**	68.60%±0.46%	69.03%±0.50%	72.57%±0.45%
**100 Samples**	74.34%±0.46%	74.77%±0.49%	79.42%±0.46%
**200 Samples**	80.44%±0.57%	81.35%±0.50%	86.05%±0.44%

Percentage of correct predictions with standard error using summed prediction, summed enrichment score, and merged models with varying sample sizes.

For the expression data, the predictive accuracy was similar among all model types (Table 5). For the genotype data, multi-task learning had a significantly higher predictive performance than the concatenated model for analyses with a sample size of 50 or more (Table 6). However, multi-task learning did not perform better than single-task learning for any of the sample sizes (Table 6). For the models that utilize matched data, the merged model had a significant improvement in predictive accuracy compared to the best unmatched model for all sample sizes (Table 7). The summed prediction and summed enrichment score models also had a significantly higher predictive performance than the best unmatched model for the analysis with a sample size of 10 (Table 7). As expected, the predictive accuracy improved as the number of samples increased for all model types, but multi-task learning did not appear to benefit more than the other model types.

### Predictive performance of multi-task with varying number of tasks

**Figure 4:**
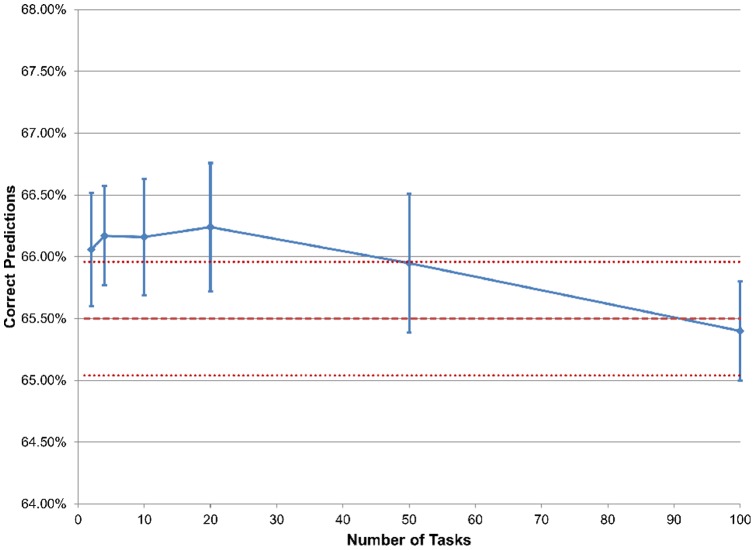
Performance of expression data with varying number of tasks. The solid line represents the change in predictive accuracy as the number of tasks changes, with the error bars being standard error. The middle dashed line represents the predictive accuracy of a single-task model with one task, with the outer dashed lines being standard error.

**Figure 5:**
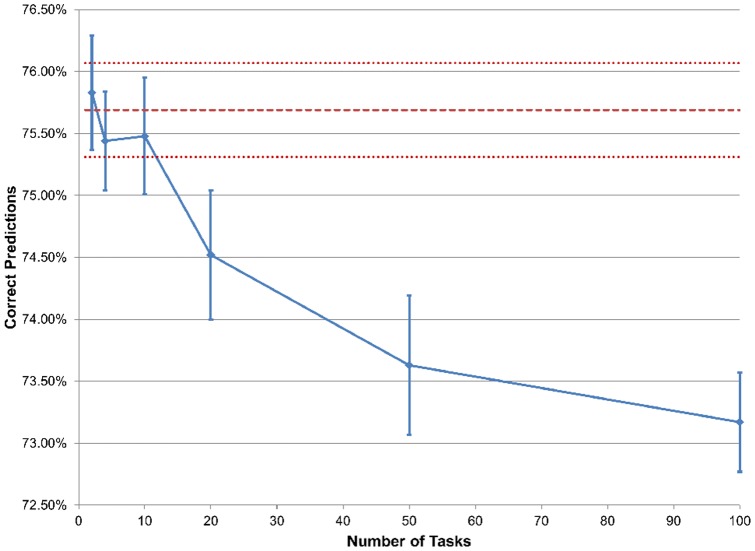
Performance of genotype data with varying number of tasks. The solid line represents the change in predictive accuracy as the number of tasks changes, with the error bars being standard error. The middle dashed line represents the predictive accuracy of a single-task model with one task, with the outer dashed lines being standard error.

We also evaluated the effect of varying the number of tasks. We generated expression data sets, each corresponding to a task, with 20 training samples evenly divided into 2 phenotypes. We simulated phenotype associated gene sets with 10 genes that were differentially expressed between the phenotypes, and background gene sets with 10 genes that represented a null model of random expression. We generated a task by simulating the first 30 gene sets as phenotype associated gene sets and the next 20 gene sets as background. For the last 50 gene sets, 30 were randomly chosen to be phenotype associated and the other 20 background. We generated additional tasks in the same way. As a result, the first 50 gene sets contained similar enrichment among all tasks, and the last 50 gene sets contained enrichment unique to each task. We used this data to build multi-task models with the number of tasks used to build each model varying from 2 to 100. We also used this data one task at a time to build single-task models for comparison. After using the 20 training samples for each task to train the model, we used 20 test samples for each task to obtain predictions. We repeated to obtain 10,000 predictions for each number of tasks and determined the percentage of correct predictions for each number of tasks (Figure 4). We also performed the same analysis with simulated genotype data (Figure 5). For the genotype data, phenotype associated gene sets contained genes that were genetically associated and background gene sets contained genes that were not genetically associated.

For the expression data, the predictive accuracies of all multi-task models and of the single-task model showed no significant difference (Figure 4). For the genotype data, the predictive performance of the multi-task experiments with 10 tasks or less was not significantly different than the single-task model (Figure 5). However, multi-task models with 20 tasks or more had a significantly lower predictive accuracy than the single-task model (Figure 5). Although this suggests that analyses with a large number of tasks may have a significant difference in performance between multi-task learning and single-task learning, most analyses of biological data will have a small number of different data types or tasks. For these data, our multi-task framework may not be significantly different than single-task in terms of predictive accuracy.

### Finding pathways enriched across multiple data types

In addition to being used for class prediction of unknown samples, we can analyze the trained models to determine whether this integrative approach provides an improved ability to discover gene sets enriched across multiple data types. A predictive weight for each gene set can be derived from the predictive model that results from an SVM analysis (see Methods). Gene sets with higher weights contribute more to prediction, and also may be more important in distinguishing phenotype. After training a multi-task SVM model, a common weight can be derived that is interpreted as a measure of importance for prediction derived from all tasks (see Methods). Gene sets with larger common weights can be viewed as sharing common information important for prediction across all tasks. These gene sets may represent biological pathways with important factors in multiple data types that are influencing phenotype. We designed the following simulation to determine the ability of our framework to discover this type of gene set.

We simulated matched expression and genotype data for 400 samples that were evenly divided into 2 phenotypes. This data contained the same gene set types as the first simulation study. We created this data set with 1 of gene set type 1 (the target gene set), 5 of gene set type 2, 5 of gene set type 3, and 89 of gene set type 4. We wanted to test the ability of our framework to extract the one target gene set, which contains genes that are both differentially expressed and genetically associated. We used our framework to train multi-task, single-task, and concatenated data predictive models. For multi-task, we determined the rank of the common weight in the predictive model for the target gene set. For single-task, we calculated the rank of the weight in the predictive model for the target gene set in the expression model and the genotype model separately. For the concatenated data model, we calculated the rank of the target gene set in a single predictive model built by concatenating the enrichment scores of the expression and genotype data. We also took the sum of the weights for both single-task models and determined the rank of the combined weight for the target gene set. In addition, we trained the summed enrichment score and merged predictive models. For the summed enrichment score model, we calculated the rank of the target gene set in a single predictive model built by taking the sum of the enrichment scores from the expression and genotype data. For the merged model, we built a single predictive model by taking the enrichment scores from the expression and genotype data and merging them into a single feature vector for each sample. We then took the sum of the expression and genotype weights for each gene set and determined the rank of the combined weight for the target gene set. We repeated this analysis 1000 times and calculated the average rank for each model type (Table 8).

**Table 8:** Average rank of target gene set.

	Average Rank
**Single-Task Expression Weight**	11.01±0.47
**Single-Task Genotype Weight**	4.17±0.10
**Single-Task Weights Summed**	3.07±0.16
**Multi-Task Common Weight**	3.08±0.16
**Concatenated Weight**	2.89±0.14
**Summed Enrichment Score Weight**	3.54±0.11
**Merged Weights Summed**	2.87±0.10

Average rank with standard error in single-task, multi-task, concatenated, summed enrichment score, and merged models for a gene set containing genes that are both differentially expressed and genetically associated with phenotype.

The average rank for the target gene set was significantly lower in all models that considered both tasks (single-task summed, multi-task, concatenated, summed enrichment score, and merged) compared to either of the separate single-task models (Table 8). This suggests that an integrated approach may be beneficial for discovering biological pathways that have an important effect within several genomic data types.

### Analysis of breast cancer data set

To provide support for the usefulness of our framework in identifying phenotypically relevant pathways, we applied it to matched expression and genotype data for breast invasive carcinomas (BRCA) generated through The Cancer Genome Atlas (TCGA) project. This data set contained 61 samples that were classified as estrogen receptor (ER) negative and 203 samples that were ER positive. Our collection of gene sets was compiled from the curated canonical pathways in the Molecular Signatures Database (MSigDB) [16]. We filtered these gene sets to only include those with 15 to 100 mapped genes, resulting in 538 gene sets.

First, we wanted to determine the ability of the data to predict ER status. We performed leave-one-out (LOO) cross-validation to calculate predictive accuracy for multi-task, single-task, concatenated, summed prediction, summed enrichment score, and merged models (Tables 9, 10, and 11). For the expression data, the predictive performance was very high for all model types, both with respect to overall accuracy and positive and negative predictive values (Table 9). For the genotype data, the overall predictive performance was moderate, but the negative predictive value (NPV) was low (Table 10). For the models that utilize matched data, the predictive accuracy was moderately better than using the genotype data alone, but not better than using the expression data alone (Table 11). The negative predictive value was greatly improved for the models that utilize matched data when compared to using the genotype data alone (Table 11). These results suggest that important gene sets in the predictive models may be biologically relevant to ER status.

**Table 9:** Correct predictions for breast cancer expression data.

	Single-Task	Multi-Task	Concatenated
**Overall Accuracy**	92.42% (244/264)	92.05% (243/264)	92.05% (243/264)
**Positive Predictive Value**	94.63% (194/205)	95.05% (192/202)	95.05% (192/202)
**Negative Predictive Value**	84.75% (50/59)	82.26% (51/62)	82.26% (51/62)

Percentage of correct predictions for the breast cancer expression data using single-task, multi-task, and concatenated models.

**Table 10:** Correct predictions for breast cancer genotype data.

	Single-Task	Multi-Task	Concatenated
**Overall Accuracy**	77.65% (205/264)	78.41% (207/264)	78.79% (208/264)
**Positive Predictive Value**	81.03% (188/232)	81.74% (188/230)	82.10% (188/229)
**Negative Predictive Value**	53.13% (17/32)	55.88% (19/34)	57.14% (20/35)

Percentage of correct predictions for the breast cancer genotype data using single-task, multi-task, and concatenated models.

**Table 11:** Correct predictions using matched data models for breast cancer data.

	Summed Prediction	Summed Enrichment Score	Merged
**Overall Accuracy**	85.98% (227/264)	88.26% (233/264)	83.71% (221/264)
**Positive Predictive Value**	86.40% (197/228)	89.81% (194/216)	85.40% (193/226)
**Negative Predictive Value**	83.33% (30/36)	81.25% (39/48)	73.68% (28/38)

Percentage of correct predictions for the breast cancer data using summed prediction, summed enrichment score, and merged models.

We next examined gene sets with the highest weights in the predictive models for their biological relevance to ER status. To allow for a direct comparison of the predictive weights among gene sets, we first normalized the enrichment scores from ASSESS (see Methods). We calculated the ranks of all gene sets for multi-task, single-task, concatenated, summed enrichment score, and merged models. A complete list of all ranks and weights for all gene sets and model types is presented in Tables S1 and S2. It is interesting to note that the ranks vary considerably among all models types. This includes significant differences between the multi-task model that considers all data simultaneously and the expression single-task and genotype single-task models which consider only data from one data type. This suggests that using an integrative approach provides results distinct from analyses of either data type alone.

A list of the top 15 gene sets with the highest common weight in the multi-task model is presented in Table 12, along with the corresponding rank of the gene sets in the expression single-task and genotype single-task models. The common weight from the multi-task model can be interpreted as the importance in distinguishing phenotype drawn from both tasks simultaneously, whereas the ranks in the single-task models provide a way to estimate the contribution that each data type had in the overall integrated rank of the gene set. An analysis of the top 15 gene sets from the multi-task model showed they were related to estrogen, steroids, cell signaling, or the cell cycle, discussed in more detail below. This provides support for the usefulness of our framework in identifying pathways associated with complex traits.

**Table 12:** Top gene sets in breast cancer analysis.

	Multi-Task Common Weight Rank	Expression Single-Task Weight Rank	Genotype Single-Task Weight Rank
**HER2 Pathway**	1	1	368
**Phase II Conjugation**	2	11	6
**Steroid Hormone Biosynthesis**	3	7	29
**FRS2-Mediated Cascade**	4	10	26
**One Carbon Pool by Folate**	5	2	152
**Neurotransmitter Release Cycle**	6	53	3
**Nitrogen Metabolism**	7	3	225
**Steroid Biosynthesis**	8	23	13
**Cholesterol Biosynthesis**	9	21	34
**Apoptotic Signaling in Response to DNA Damage**	10	19	131
**Riboflavin Metabolism**	11	35	12
**ECM-Receptor Interaction**	12	164	1
**Nuclear Receptor Transcription**	13	50	18
**Mitotic Prometaphase**	14	121	4
**Steroid Metabolism**	15	8	154

Gene sets with the largest multi-task common weights in the breast cancer analysis, along with the ranks of the expression and genotype single-task weights.

Estrogen plays an important role in breast cancer [28]. We found that three of the top 15 gene sets were directly related to estrogen signaling and metabolism: “HER2 Pathway” (rank 1), “Phase II Conjugation” (rank 2), and “Nuclear Receptor Transcription” (rank 13). Human epidermal growth factor receptor 2 (HER2), encoded by the gene *ERBB2*, influences the expression and activity of the estrogen receptor [29]. The “HER2 Pathway” gene set contains the estrogen receptor 1 (*ESR1*) gene. The “Nuclear Receptor Transcription” gene set also contains the *ESR1* gene, and nuclear receptor coactivators are thought to participate with the estrogen receptor pathway [30]. Several phase II conjugating enzymes are involved with the metabolism of estrogen [31]. Tamoxifen is an antiestrogenic drug that is widely used in the treatment of ER positive breast cancer [32]. One study showed that genetic variation in several phase II conjugating enzymes influenced the efficacy of Tamoxifen therapy in breast cancer [33]. Since this study linked genotype differences to Tamoxifen efficacy, it is interesting to note that the Phase II Conjugation gene set has the sixth highest genotype single-task weight (Table 12) and is the highest ranked gene set in the multi-task genotype model (w2, Table S1). It is also the eleventh highest gene set in the single-task expression model (Table 12) and has the fourth highest rank in the multi-task expression model (w1, Table S1). This suggests that genotype differences may be directly influencing expression changes. The strong association in both the expression and genotype data resulted in the second highest rank in the multi-task common weights (Table 12), which is higher than the weight in either of the single-task models alone. All three of the estrogen-related gene sets contained genes that were generally overexpressed in the ER positive samples.

Estrogen is a steroid hormone, and we found that four of the top 15 gene sets were involved with the synthesis or metabolism of steroids: “Steroid Hormone Biosynthesis” (rank 3), “Steroid Biosynthesis” (rank 8), “Cholesterol Biosynthesis” (rank 9), and “Steroid Metabolism” (rank 15). In addition to estrogen, other steroid hormones, such as progesterone, play an important role in breast cancer [28]. Also, many steroids, including estrogen, are synthesized from cholesterol, and one study showed that cholesterol levels are linked with breast cancer prognosis [34].

The estrogen receptor participates in cellular signaling initiated by the binding of estrogen and facilitating the activation of downstream processes. In addition to the estrogen-related pathways, three of the top 15 gene sets were similarly involved with other types of cell signaling: “FRS2-Mediated Cascade” (rank 4), “Neurotransmitter Release Cycle” (rank 6), and “ECM-Receptor Interaction” (rank 12). The FRS2-mediated cascade links Fibroblast Growth Factor Receptor (FGFR) to the eventual activation of several important signaling pathways. One study showed that blocking FGFR inhibited breast cancer proliferation and led to downregulation of the MAPK and PI3K pathways [35]. Also, ECM receptors may participate in the control of many stages of breast cancer [36], and neurotransmitters may influence the metastasis of breast tumors [37]. All three of these cell signaling gene sets contained genes that were generally overexpressed in the ER positive samples.

Tumors accumulate genetic damage that results in a perturbed cell cycle which increases the number of tumor cells by stimulating cell birth or inhibiting cell death or cell-cycle arrest [12]. Many of the previously discussed gene sets are involved with the cell cycle or metabolism, and we found that the five remaining gene sets in the top 15 were also involved with the cell cycle and metabolism: “One Carbon Pool by Folate” (rank 5), “Nitrogen Metabolism” (rank 7), “Apoptotic Signaling in Response to DNA Damage” (rank 10), “Riboflavin Metabolism” (rank 11), and “Mitotic Prometaphase” (rank 14). Disrupting mitotic prometaphase may influence cell-cycle arrest, and disrupting apopototic signaling in response to DNA damage may inhibit the cell death of tumor cells. Also, folate, nitrogen, and riboflavin, also known as vitamin B2, are important for cell growth. One study linked increased consumption of folate and B vitamins with reduced risk of breast cancer [38].

## Discussion

Although the simulation study showed that our integrative framework provided an improved ability to discover pathways that are enriched over multiple data types, multi-task learning performed about the same as similar integrated learning methods. Also, the predictive accuracy of multi-task learning was practically the same as similar learning methods. All of these results suggest that while an integrated pathway approach may be useful for discovering relevant pathways, it may not be necessary to use multi-task learning for most studies. Further research should explore alternative prediction methods.

This study focused on the integration of gene expression and genotype data. However, our framework may also be suitable for other genomic data types, such as copy number variation and DNA methylation. Also, the sample-specific enrichment scores from multiple data types can be used for many sample-level pathway-based analyses, such as clustering to find subtypes of samples with similar pathway enrichment profiles.

Results from this study indicate that a pathway-based integrative analysis is a promising approach to identify pathways that are influenced by both gene expression changes and genotype variation. All of the top 15 pathways from the multi-task model built using breast cancer data have been previously associated with breast cancer. This suggests that an integrative approach may be useful for discovering pathways related to complex diseases, especially diseases that are not as well understood, and for determining the contribution that each data type has for each pathway. The “Phase II Conjugation” gene set is an example that had a strong association in both the expression and genotype data, and this gene set had the second highest multi-task common weight, which was higher than in either of the single-task models alone. This supports the use of an integrative approach in discovering gene sets that may have a direct link between genotype and expression.

## Materials and Methods

Our integrative framework contains two keys steps: 1) pathway enrichment analysis using ASSESS and 2) building a predictive model using an SVM. This framework is designed for integrating different genomic data types into a predictive model for samples that have been designated into one of two phenotypic classes.

### Pathway Enrichment

#### ASSESS

To perform the gene set analysis step of our framework, we used a software package called ASSESS [22]. ASSESS takes gene-based genomic data along with phenotype information and provides a measure of the variation of gene set enrichment over all samples for a given gene set. First, ASSESS computes a correlation statistic for each sample and gene as
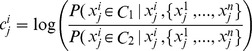
where 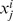 is data for the *i*-th sample and *j*-th gene, and *C_1_* and *C_2_* are the two phenotypic classes. Next, ASSESS independently uses the correlation statistics for each sample to compute enrichment scores for each gene set using a weighted Kolmogorov-Smirnov statistic. The original implementation of ASSESS includes two metrics for calculating correlation statistics for expression data.

#### Normalizing the Enrichment Scores

To normalize the original enrichment scores, we permuted the class labels and recalculated new background enrichment scores 1000 times. If the original enrichment score for a sample and gene set was positive, this score was divided by the average of the positive background enrichment scores for that sample and gene set. If the original enrichment score was negative, this score was divided by the absolute value of the average of the negative background enrichment scores.

#### Extension of ASSESS for Genotype Data

To calculate gene set enrichment scores for genotype data, we extended ASSESS. The first step is associating SNP-based genotype data with genes. To do this, we first identify all SNPs that are within a pre-defined distance surrounding and including a given gene. Then, we use Person's chi-square test to determine the extent to which each SNP correlates with phenotype. Finally, we select the SNP that has the maximum correlation with phenotype as the “representative” SNP for that gene. After obtaining gene level data, each correlation statistic is calculated as
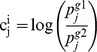
where 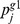 is the percentage of samples with the genotype of the *i*-th sample for the *j*-th gene in class 1, and 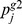 is the percentage of samples with this genotype in class 2. If either class contains zero samples with a given genotype, a pseudo-count of 1 is added. These correlation statistics are then used to obtain enrichment scores in the same way as ASSESS.

### Predictive Model

#### SVM Framework

To perform the predictive modeling step of our framework, we used a software package called SVM-Light [39]. All single-task and concatenated analyses use a standard linear kernel. The SVM trains a predictive model by calculating nonnegative Lagrange multipliers for each sample, *α_i_*. These sample weights are used to derive predictive weights for each gene set as
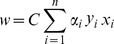
where *C* is a regularization parameter, *y_i_* is the class assignment of the *i*-th sample and *x_i_* is data for the *i*-th sample.

#### Multi-Task SVM

To utilize a multi-task framework, we used regularized multi-task learning [25], which is an implementation of an SVM that incorporates multi-task learning. We used SVM-Light with the following custom linear kernel:
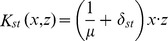
where *μ* is a positive parameter that controls the relatedness of the models, and *δ_st_* = 1 if *s* and *t* belong to the same task, *δ_st_* = 0 otherwise. The SVM trains a predictive model by calculating nonnegative Lagrange multipliers for each sample and task, 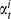. These sample weights are used to derive task-specific effects for each gene set and task as
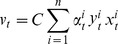
where *C* is a regularization parameter, 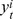 is the class assignment of the *i*-th sample in task *t*, and 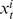 is data for the *i*-th sample in task *t*. These weights are used to calculate common weights for each gene set as
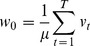
where *T* is the number of tasks. The common weights are summed with the task-specific effects for each gene set and task to compute the final predictive weights.

### Simulated Data

We simulated data similar to a previous study that integrated gene expression and genotype data for pathway analysis [40]. For analyses using matched data, the genotype used to generate the expression value for a sample was used as the genotype data for that sample.

#### Genotype

Each genotype data set contained genes that were either genetically associated or had a random genotype. We mapped genes that were genetically associated to a single causal SNP, and we mapped genes that had a random genotype to a single random SNP. We simulated the causal SNPs based on parameters estimated from genotype information for glioblastoma generated through The Cancer Genome Atlas (TCGA) project [11]. We based these SNPs on the P53PATHWAY, as defined in version 2.5 of the Molecular Signatures Database (MSigDB) [16]. First, we mapped a single SNP in the glioblastoma data to each of the genes in the P53PATHWAY. To do this, we found the SNPs within the region 1,000 bases upstream of the transcription start site to the end of the transcribed region of each gene. Then, we selected the SNP with minor allele frequency greater than 0.05 that had the highest chi-square association with glioblastoma. We set the allele frequencies of the causal SNPs in the simulated data to that of these selected SNPs in the glioblastoma data. We generated the heterozygote odds ratio for each SNP from U[1.1,1.3] and used an additive disease model with a disease prevalence of 0.02. Using these parameter settings, we generated genotype data using PLINK [41]. We determined the probability that each sample belongs to class 1 based on the following model:
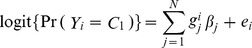
where *N* is the number of causal SNPs, 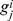 is the coding of the genotype of the *i*-th sample for the *j*-th SNP, *β_j_* is the log of the heterozygote odds ratio for the *j*-th SNP, and *e_i_* is an error term for the *i*-th sample drawn from a standard normal distribution. We randomly assigned each sample to either class 1 or class 2, with the probability of being assigned to class 1 equal to the probability calculated in the model above. We also generated random genotype data using PLINK. For the random genotype data, we drew allele frequencies from Beta(0.1,0.1) and assigned a heterozygote odds ratio of 1.

#### Gene Expression

Each gene expression data set contained genes that were either differentially expressed or had random expression. We simulated the expression data based on the TCGA glioblastoma study. We based genes that were differentially expressed on the P53PATHWAY. We calculated the mean vector *μ* and the covariance matrix *Σ* of the genes in the P53PATHWAY. We used this to generate baseline expression levels by drawing from a multivariate normal distribution, *X_0_*∼N(*μ*,*Σ*). We added a disease effect to these genes by linking each gene to a causal SNP and calculated the final expression level as
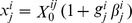
where 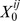 is the baseline expression of the *i*-th sample for the *j*-th gene, 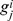 is the coding of the genotype of the *i*-th sample for the *j*-th SNP, and 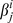 is the effect size of the genotype on gene expression that is drawn from U[1.0,1.5]. We also generated random expression data. We calculated the mean of all genes in the glioblastoma data and took the average of these means as *μ_0_* and determined the standard deviation of all genes and the average as *σ_0_*. We used these parameters to generate random expression levels by drawing from a normal distribution, *X*∼N(*μ_0_*, *σ_0_* ^2^).

### Breast Cancer Data

We obtained breast invasive carcinoma (BRCA) data generated through The Cancer Genome Atlas (TCGA) project from their data portal (http://cancergenome.nih.gov). We selected samples that provided matched gene expression and genotype data. We filtered samples to only include patients who were white, female, 40 to 70 years of age at initial diagnosis, and had a known estrogen receptor (ER) status of positive or negative. We also eliminated the sample with barcode “TCGA-A2-A0CY” because of unreliable genotype data. This resulted in a data set of matched gene expression and genotype data for 61 ER negative samples and 203 ER positive samples.

## Supporting Information

Table S1 **Rank of predictive weights in breast cancer analysis.** Rank of the predictive weights for the breast cancer data using multi-task, single-task, concatenated, summed enrichment score, and merged models.
(XLSX)

Table S2 **Predictive weights in breast cancer analysis.** The predictive weights for the breast cancer data using multi-task, single-task, concatenated, summed enrichment score, and merged models.
(XLSX)
