# Correction: A Predictive Framework for Integrating Disparate Genomic Data Types Using Sample-Specific Gene Set Enrichment Analysis and Multi-Task Learning

**DOI:** 10.1371/annotation/c04cea96-35f4-4578-a891-639e30fddd59

**Published:** 2013-09-09

**Authors:** Brian D. Bennett, Qing Xiong, Sayan Mukherjee, Terrence S. Furey

The authors wish to publish the following erratum for the Acknowledgements Statement: "The results published here are in whole or part based upon data generated by The Cancer Genome Atlas pilot project established by the NCI and NHGRI. Information about TCGA and the investigators and institutions who constitute the TCGA research network can be found at http://cancergenome.nih.gov/. The TCGA data analyzed here are available through dbGAP, accession phs000178.v8.p7 (http://www.ncbi.nlm.nih.gov/projects/gap/cgi-bin/study.cgi?study_id=phs000178.v8.p7)."

